# Neuroprotective activity of a new erythropoietin formulation with increased penetration in the central nervous system

**DOI:** 10.1186/1753-6561-5-S8-P3

**Published:** 2011-11-22

**Authors:** Marina Etcheverrigaray, Natalia Ceaglio, Mónica Mattio, Marcos Oggero, Ignacio Amadeo, Guillermina Forno, Norma Perotti, Ricardo Kratje

**Affiliations:** 1 Cell Culture Laboratory, School of Biochemistry and Biological Sciences, Universidad Nacional del Litoral. Ciudad Universitaria – C.C.242 – (S3000ZAA) Santa Fe, Provincia de Santa Fe, Argentina; 2 Zelltek S.A. Ruta Nacional 168 – PTLC – (3000) Santa Fe, Provincia de Santa Fe, Argentina

## Background

Apart from its hematopoietic effect, erythropoietin (EPO) is a molecule with high neuroprotective potential. However, its prolonged application may cause serious adverse effects due to the erythropoiesis stimulation. Therefore, an EPO derivative with neuroprotective properties but low hematopoietic activity, designated as neuropoietin (rhNEPO), was developed in our lab using an alternative purification process of the recombinant human erythropoietic counterpart (rhEPO) produced in CHO cells [1]. The *in vitro* cytoprotective activity of rhNEPO on neural phenotype cells and its brain uptake from blood are herein analyzed.

## Results

*In vitro* citoprotective activity of rhNEPO was analyzed on rat pheochromocytoma cells (PC-12) differentiated to neural phenotype with neural growth factor (NGF). Apoptosis was triggered by NGF and serum withdrawal from cell cultures. Thus, nuclear DNA fragmentation was analyzed by colorimetric TUNEL detection. One-way analysis of variance was carried out followed by Dunnett´s multiple comparison test. Probabilities lower than 0.05 were considered significant (p<0.05).

**Figure 1:**
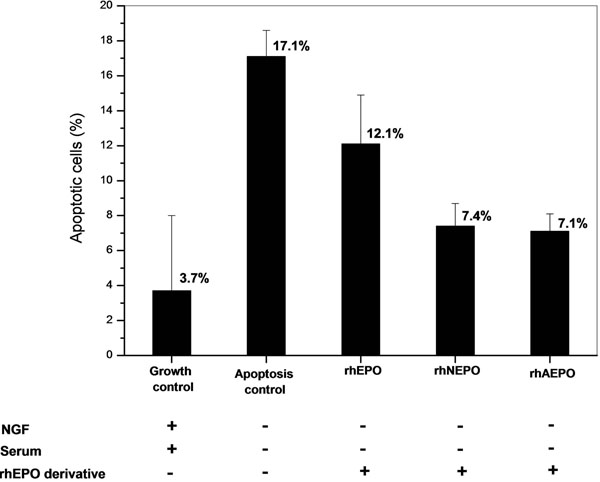
Protective effect of rhEPO derivatives on differentiated PC-12 cells upon induction of apoptosis by NGF and serum withdrawal. rhEPO, rhNEPO and desialylated rhEPO (rhAEPO) were added to the culture 24 h prior to induction of cell death. Apoptosis was measured by TUNEL technique.

As shown in Figure 1, serum and NGF withdrawal significantly increased the number of apoptotic cells to 17.4 ± 1.5% (apoptosis control) compared to 3.7± 4.3% from growth control (p<0.05). rhEPO slightly protected differentiated PC-12 cells from death since apoptotic cells decreased to 12.1 ± 2.8% compared to the apoptosis control. Interestingly, rhNEPO and desialylated rhEPO (an EPO derivative where sialic acid were enzimatically removed) completely protected cells from apoptosis as both analogues showed 7.4 ± 1.3% and 7.1 ± 1.0% of apoptotic cells respectively (p<0.05). Besides, none of the derivatives exhibited significant differences with the growth control (p>0.05), confirming their properties to protect PC-12 cells from apoptosis. Therefore, this novel combination of erythropoietin glycoforms (rhNEPO) with lower sialic acid content and antennarity than rhEPO [1] preserved its binding receptor capacity exerting an *in vitro* neuroprotective activity even better than the mentioned counterpart. Also, rhAEPO showed an in vitro activity that is similar to that of rhNEPO, having both derivatives the lowest content of carbohydrates. It is well known that the affinity of EPO analogues for EPO receptor is inversely related to the sialylation of their attached carbohydrate [2] and that removal of sialic acid turns it into a molecule with a very short half-life with almost no erythropoietic activity. This is the case of rhAEPO that explains its rapid hepatic clearance from blood [3]. For that reason, rhNEPO emerges as a neuroprotective candidate displaying higher *in vitro* activity than rhEPO.

Taking into account the cytoprotective activity of rhNEPO on neural phenotype cells, cerebrospinal fluid (CSF) and blood pharmacokinetics of rhEPO and rhNEPO were evaluated in rats following intravenous administration of a single dose of each protein, aiming to evaluate their CSF uptake from plasma.

The distribution and the elimination half-lives of rhNEPO in blood were significantly shorter than the corresponding ones for rhEPO. Differences in the sialic acid content and oligosaccharide anntenarity status [1] may describe the faster elimination rate of rhNEPO. Thus, this derivative, which is a less sialylated and less branched molecule, was rapidly cleared from blood but reached the CSF in a shorter time (5 min vs 30 min of rhEPO) at concentrations high enough to bind to the EPO receptors. Consequently, the faster transport through the blood-brain-barrier (BBB) might accelerate rhNEPO distribution into the nervous system, causing a faster appearance into the action site. Moreover, the rapid clearance of rhNEPO from plasma represents an advantage in the treatment of neurological diseases, because the continuous presence of EPO in blood is the stimulus that triggers the production of sanguineous cells with the resulting appearance of adverse side-effects.

## Conclusions

The *in vitro* anti-apoptotic effect of rhNEPO becomes a remarkable fact to predict its neuroprotective action in the nervous system. Furthermore, despite its short plasma half-life, rhNEPO appears promptly within the CSF, being also a further and a significant fact that encourage the study of neuroepoetin as a potential drug for the treatment of neurological diseases, in which, a highly neuroprotective activity with low side effects and a fast blood-to-brain influx are desirables.

**Table 1:** Pharmacokinetics of rhEPO and rhNEPO in plasma and CSF after intravenous administration of a single dose of 500 µg of each protein in rats. The quantification of EPO derivatives were carried out by sandwich ELISA [1].

Pharmacokinetics		rhEPO			rhNEPO		
**Plasma ^1^**	*t_1/2α_* (*h*) *^a^*	1.7	±	0.1	0.4	±	0.1
	*t_1/2β_* (*h*) *^b^*	12.3	±	1.0	8.9	±	0.4
**CSF ^2^**	*t_CSF_*_(_*_min_*_)_*^c^*	30			5		

^a^ t_1/2α_, distribution half-life; ^b^ t_1/2β_, elimination half-life; ^c^ time to reach CSF after administration of EPO derivatives. ^1^Distribution and elimination half-lives and their corresponding standard deviations were obtained using a bi-compartmental pharmacokinetic model. ^2^ The *t_CSF_* refers to the first time point in which EPO derivatives were detected in CSF, and for this reason standard deviations were not considered.

## References

[B1] Mattio M Ceaglio N Oggero M Perotti N Amadeo I Orozco G Forno G Kratje R Etcheverrigaray M Isolation and characterization of a subset of erythropoietin glycoforms with cytoprotective but minimal erythropoietic activity Biotechnol Progr 2011 doi: 10.1002/btpr.633 10.1002/btpr.633 21608141

[B2] Egrie JC Browne JK Development and characterization of novel erythropoiesis stimulating protein (NESP) Nephrol Dial Transplant 2001 16 Suppl 3 3 13 11402085

[B3] Fukuda MN Sasaki H Lopez L Fukuda M Survival of recombinant erythropoietin in the circulation: the role of carbohydrates Blood 1989 73 84 89 2910371

